# Serological Survey on the Occurrence of Anti-*Leptospira* spp. Antibodies in Red-Eared Terrapins (*Trachemys scripta elegans*) Living in a Natural Park of Northern Italy

**DOI:** 10.3390/ani11030602

**Published:** 2021-02-25

**Authors:** Eleonora Bonacina, Maurizio Oltolina, Roberto Robbiati, Paolo Pinzauti, Valentina Virginia Ebani

**Affiliations:** 1 Parco Faunistico Le Cornelle, via Cornelle 16, 24030 Valbrembo (BG), Italy; bonacina@lecornelle.it (E.B.); oltolina@lecornelle.it (M.O.); robbbiati.r@gmail.com (R.R.); 2 Department of Veterinary Science, University of Pisa, viale delle Piagge 2, 56124 Pisa (PI), Italy; paolo.pinzauti@unipi.it

**Keywords:** *Leptospira*, Tarassovi, *Trachemys scripta elegans*, zoonosis, antibodies, chelonians

## Abstract

**Simple Summary:**

Although it has been supposed that reptiles, including chelonians, are involved in the epidemiology of *Leptospira*, data about leptospirosis in turtles are very scant. In the present survey, *Trachemys scripta elegans* terrapins living in a natural park were tested to verify the presence of antibodies against *Leptospira* spp., with 6% being positive. Infected turtles could be a source of infection for other animals and humans, shedding leptospirae into the environment. However, further studies are necessary to verify if *Leptospira* may cause disease in turtles or if these animals may serve as reservoirs.

**Abstract:**

Turtles are suspected to be involved in the epidemiology of *Leptospira*; however, data about the dissemination of this zoonotic pathogen among chelonians are scant. In the present study, the serum samples collected from 49 *Trachemys scripta elegans* living in a natural park of northern Italy were tested by a microagglutination test to measure detectable antibodies against different *Leptospira* serovars. Three (6.12%) turtles had agglutinins to the serovar Tarassovi, suggesting that they were exposed to the spirochaetes. Currently, it is not clear if *Leptospira* can cause disease in chelonians or if these animals can serve as reservoirs of leptospirae. Considering that chelonians often share the same environment with other animals and humans, and considering the One Health perspective, investigations to better understand the role of chelonians as a source of *Leptospira* infection are necessary.

## 1. Introduction

*Leptospira* is a spirochaetal bacterium responsible for severe disease in humans and several animal species. Its dissemination is related to different environmental features, surviving best in humid areas and in the presence of animals acting as accidental or maintenance hosts [1,2].

Several domestic and wild mammal species have been found to be a natural reservoir or accidental hosts for different serovars of *Leptospira* spp. [3]. On the other hand, data about leptospirosis in poikilothermic animals are limited. In the past few decades, increasing attention has been turned to the role of reptiles in the epidemiology of leptospirosis. Currently, it is not clear if leptospirae can induce disease in poikilothermic animals or if they are important in the epidemiology as urinary shedders of leptospirae. Understanding this aspect of transmission is important because reptiles could contribute to the dissemination of leptospirae in the environment and serve as a source of infection for other animals and humans.

A few studies have been carried out to detect specific antibodies and/or leptospirae in specimens collected from reptiles [4,5,6,7,8]. Chelonian species have been of particular interest, especially aquatic species, because of the humid environment where they live [5,6,9,10,11].

The red-eared terrapin *Trachemys scripta elegans* (*T. scrpta elegans*) is a semiaquatic turtle that is very popular as a pet animal in many parts of the world. Furthermore, it is considered an invasive species and is often abandoned near rivers, lakes and natural or public ponds when the terrapins grow out of their enclosure or their caretaker tires of them [12].

Past surveys found high seroprevalence values among terrapins [7,8]. A recent study carried out in Italy found *T. scripta elegans* and *T. scripta scripta* seropositive to different *Leptospira* serovars [10].

Unfortunately, there have been only a limited number of studies characterizing the role of terrapins in the epidemiology of leptospirosis. More information about leptospiral exposure in *T. scripta* is needed to better understand the role of terrapins in the epidemiology of this zoonotic disease.

The aim of the present study was to evaluate the occurrence of antibodies to different serovars of *Leptospira* spp. in *T. scripta elegans* terrapins living in a natural park in northern Italy.

## 2. Material and Methods

### 2.1. Animals

A total of 49 terrapins, *T. scripta elegans*, were sampled in this study. One hundred and twenty-eight terrapins were present in the natural Park “Le Cornelle” located in Valbrembo, Bergamo province (northern Italy; 45°42′57.29″ N–9°35′53.91″ E). However, the samples were collected only from the terrapins that had lived in the park for more than ten years. All turtles lived in a closed area in which a pond was present. No contact was possible between turtles and other animals hosted in the park, but wild animals (birds, rodents, lizards) could have access to the area.

Turtles were fed with commercial thawed fish twice a week, except for the period from October to March when they were fasting. Turtles were submitted to coprological examinations every four months and, when necessary, treated with specific antiparasitic drugs.

At the sampling time (April 2019), each terrapin was carefully examined by veterinarians who verified that all animals were in good health and showed no apparent signs of disease.

### 2.2. Microagglutination Test

Whole-blood samples were collected from the cervical vertebral venous plexus of each animal. Sera were separated by centrifugation at 1300× *g* for 10 min and stored at −20 °C until tested.

All samples were tested by microagglutination test (MAT) using the Martin and Pettit test to measure antibodies against *Leptospira* spp. Live cultures of the following 8 serovars were used as antigens: Ballum (strain Ballico), Bratislava (strain Riccio 2), Canicola (strain Alarik), Grippotyphosa (strain Moskow V), Hardjo (strain Hardjoprajitno), Icterohaemorrhagiae (strain Bianchi), Pomona (strain Mezzano), Tarassovi (strain Johnson). The selected serovars represent a spectrum that was expected to be prevalent in Italy. Antigens were collected from 4–14 day cultures, containing 1–2 × 10^8^ leptospires/mL, grown in Leptospira Medium Base Ellinghausen-MacCullough–Johnson–Harris (EMJH—Difco, Becton, Dickinson and Company, Sparks, MD, USA) at 30 °C and checked for purity, mobility and agglutination power [13].

Sera were diluted 1:25 with sterile saline solution in wells of 96 U-shaped plates. To obtain a 1:50 final dilution, considered as the cut-off value, the same volume of the antigen suspension was added to each well and mixed by agitation. Plates were incubated at 30 °C for two hours. A loopful of the suspension in each well was placed on a slide and examined for agglutination using a dark-field microscope. Sera were considered positive when agglutination was greater than or equal to 50%.

Positive sera were successively two-fold serially diluted and tested to determine the endpoint titre.

## 3. Results and Discussion

Among the 49 tested animals, 3 (6.12%, 95% confidence interval: 0.01–12.7) had agglutinins to the serovar Tarassovi with 1:100 antibody titer. The analyses were carried out with MAT, which is the standard serological test for leptospirosis and has been the most employed in studies with reptiles [14].

Serovar Tarassovi is able to infect several animal species. While it is traditionally related to leptospirosis in farm animals (e.g., swine), other mammals have also been found to become infected by this serovar and may serve as accidental or maintenance hosts [15]. Agglutinins to the serovar Tarassovi have been found in different mammal species in northern Italy, with percentages variable in relation to the tested animals and their ecosystem [16,17].

Tarassovi has also been found in chelonians. An investigation carried out by Glosser et al. [8] found that 96% of turtles tested were serologically positive to *Leptospira* sp. The turtles sampled in the study included *T.* (formely *Pseudemys) scripta elegans, Chelydria serpentina* (common snapping turtle) and *Sternothaerus odoratus* (common musk turtle), and all three groups lived in settling ponds. Moreover, the serovar Tarassovi was isolated from cloacal and kidney specimens collected from *T. scripta elegans.*

More recently, Lindtener-Knific et al. [18] found antibodies to Tarassovi in one *T. scripta elegans* at 1:200 titer and three *Emys orbicularis* (European pond turtle) with 1:100, 1:800 and 1:1600 titers, respectively. In Italy, 10/16 *T. scripta elegans* were found to have antibodies against Tarassovi with titres ranging from 1:50 to 1:3200 [10].

The terrapins tested in the present survey could have been exposed to leptospirae eliminated in the environment by animals that shared the same area. Considering that the terrapins had no contact with the other animals hosted in the park, it could be supposed that free-ranging lizards (*Podarcis* sp.) or rodents, animals most often present in the area where terrapins lived, served as the main source. No data about leptospirosis in free-ranging lizards are available; however, some authors [18,19] have suggested that lizards are involved in the epidemiology of leptospirosis. A study measuring the seroprevalence for *Leptospira*, serovars Sejroe, Canicola, Bataviae and Gryppotyphosa, in *Lacerta viridis* and *Lacerta agilis* found that nearly 30% of animals were seropositive [19]. Moreover, antibodies to Tarassovi have also been measured in other lizard species [18]. In particular, *Uromastyx hardwickii* (Hardwick’s Spiny-tailed Lizard)*, Uromastyx dispar* (Sudan Spiny-tailed Lizard), *Eublepharis macularius* (Leopard Gecko) and *Iguana iguana* (Green Iguana), originating from Slovenia or imported from reptile farms in European countries, Pakistan and Mali, had titers ranging from 1:50 to 1:300 [18].

Rodents are well-known shedders of different *Leptospira* serovars, including Tarassovi. Rodents generally acquire leptospires as pups and maintain them as a chronic infection in the renal tubules, excreting bacteria in their urine throughout their life, often in increasing amounts [20,21].

Antibodies against other *Leptospira* serovars have also been measured in chelonians with different prevalences in relation to the tested species and the environment where the animals lived. Several species of turtles were studied, including *Emys orbicularis* (European pond turtle), *Emydoidea blandingii* (blanding’s turtle), *Trachemys dorbigby* (black-bellied slider), *Phrynops hilarii* (Hilaire’s toadhead turtle), *Sternothaerus odoratus* (stinkpot turtle), *Chelydra serpentine* (snapping turtle) and *Phrynops geoffroanus* (geoffroy’s side-necked turtle) [5,6,7,8,9,18]. Moreover, a recent interesting survey found agglutinins versus *Leptospira* sp. in sea turtles (*Eretmochelys imbricata* and *Chelonia mydas*) [22].

The results obtained from the different surveys suggest that there is no close relation between a given chelonian species and a serovar, but instead is likely related to the most common serovars cycling through all species in a specific ecosystem. The detection of antibodies against a given serovar depends on the presence of animals infected with the same serovar and consequently on its presence in the environment.

It has been suggested that antibody titers in turtles are related to environmental factors. In fact, high titers in aquatic species seem to be caused by long-term exposure to water-borne leptospires [7]; conversely, Abdulla and Karstad [23] assumed that low titers could be influenced by low environmental temperature that might negatively interfere with the immune system. However, correlation between the antibody titer and the carrier status has not been demonstrated; thus, low antibody titers cannot exclude the carrier role of seropositive turtles. 

Immune response of reptiles has not been well defined. It has been observed that humoral response in reptiles is slower than in mammals and may not be a function of the temperature alone [24]. In fact, Zimmerman et al. [25] observed that. *T. scripta* terrapins are able to produce polyreactive natural antibodies (Nabs) that are important for a strong innate immune response.

Seropositive reactions only indicate a previous contact/exposure of the animals with leptospirae and do not confirm that an animal has a chronic infection, which is a necessary condition that defines a reservoir of leptospirosis [14]. Detection of leptospirae in urine, using PCR or bacteriological isolation, can recognize animals that are shedders as a result of acute or chronic infection. Clinical disease (presence of symptoms and/or lesions) has not been reported in chelonians that are *Leptospira* positive. A recent study found that all tested Blanding’s turtles (Emydoidea blandingii) had anti-*Leptospira* antibodies, even though they appeared clinically healthy and showed no apparent signs of disease [11]. Some authors have speculated that this may be the result of turtles and leptospirae coevolving in aquatic environments [9].

Turtles could become reservoirs of leptospirae because of low pathogenicity of the infecting strain, low infectious dose and low specificity between the infected animal species and the *Leptospira* strain [13].

The percentage of seropositivity detected in this study is low compared with a recent investigation in Blanding’s turtles that found 100% in seropositive animals and 73% in RT-PCR positive ones [11]. This relevant difference could be related to the different ecosystems in which they resided. In fact, Blanding’s turtles came from a large preserve, whereas the *T.* *scripta* of our investigation lived in a closed area within a natural park.

## 4. Conclusions

The percentage of seropositivity detected in this study is low compared with the results obtained by other serological investigations in turtles. The detection of agglutinins anti-Tarassovi in the terrapins tested in our survey may indicate that the seroprevalence of *Leptospira* and the serovar involved are strictly related to the environment where animals reside.

Information about the mechanisms of the immune system of chelonians in *Leptospira* infection are not fully available, and even though leptospirae do not seem to cause disease in chelonians serological and molecular investigations are necessary to better understand the relation between these animals and *Leptospira* spp. In particular, it is necessary to better verify the role of terrapins and tortoises as shedders of leptospirae and their role in the epidemiology of *Leptospira*.

Chelonians living in parks and zoos, if shedders of leptospirae, could represent a source of infection for other animals. Moreover, particular attention should be given to turtles kept as companion animals. In fact, even though it is improbable that the water that turtles live in is contaminated by leptospirae and is a source of infection, purchased turtles could be shedders. In this case, owners could be at risk of infection during the cleaning procedures of cages.

## Data Availability

Data is contained within the article.

## References

[1] Sykes J., Hartmann K., Lunn K., Moore G., Stoddard R., Goldstein R. (2011). 2010 ACVIM small animal consensus statement on leptospirosis: Diagnosis, epidemiology, treatment and prevention. J. Vet. Intern. Med..

[2] Pal M., Hadush A. (2017). Leptospirosis: An infectious emerging waterborne zoonosis of global significance. Air Water Borne Dis..

[3] Azócar-Aedo L., Smits H.L., Monti G. (2014). Leptospirosis in dogs and cats: Epidemiology, clinical disease, zoonotic implications and prevention. Arch. Med. Vet..

[4] Ebani V.V. (2017). Domestic reptiles as source of zoonotic bacteria: A mini review. Asian Pac. J. Trop. Med..

[5] Silva E.F., Seyfer N., Cerqueira G.M., Leihs K.P., Athanazio D.A., Valente A.L.S., Dellagostin O.A., Brod C.S. (2009). Serum antileptospiral agglutinins in freshwater turtles from Southern Brazil. Braz. J. Microbiol..

[6] Grimm K., Mitchell M.A., Thompson D., Maddox C. (2015). Seroprevalence of *Leptospira* spp. in Blanding’s Turtles (*Emydoidea blandingii*) from DuPage County, Illinois USA. J. Herpetol. Med. Surg..

[7] Andrews R.D., Reilly J.R., Ferris D.H., Hanson L.E. (1965). Leptospiral agglutinins in sera from southern Illinois herpetofauna. J. Wildl. Dis..

[8] Glosser J.W., Sulzer C.R., Eberhardt M., Winkler W.G. (1974). Cultural and serologic evidence of *Leptospira interrogans* serotype Tarassovi infection in turtles. J. Wildl. Dis..

[9] Oliveira J.P., Kawanami A.E., Silva A.S.L., Chung D.G., Werther K. (2016). Detection of *Leptospira* spp. in wild *Phrynops geoffroanus* (Geoffroy’s side-necked turtle) in urban environment. Acta Tropica.

[10] Dezzutto D., Barbero R., Canale G., Acutis P.L., Biolatti C., Dogliero A., Mitzy M.D., Francone P., Colzani A., Bergagna S. (2017). Detection of *Leptospira* spp. in water turtle (*Trachemys scripta*) living in ponds of urban parks. Vet. Sci..

[11] Rockwell K.E., Thompson D., Maddox C., Mitchell M.A. (2019). Blanding’s turtles (*Emydoidea blandingii*) as a reservoir for *Leptospira* spp.. PLoS ONE.

[12] Global Invasive Species Database 2021 Species Profile: Trachemys Scripta Elegans. http://www.iucngisd.org/gisd/species.php?sc=71.

[13] Ebani V.V., Bertelloni F., Pinzauti P., Cerri D. (2012). Seroprevalence of *Leptospira* spp. and *Borrelia burgdorferi* sensu lato in Italian horses. Ann. Agric. Environ. Med..

[14] Fornazari F. (2017). Are reptiles reservoirs of leptospirosis? A brief discussion based on serological studies. Ecohealth.

[15] Ryan T.J., Marshall R.B. (1976). Isolation of a leptospira belonging to serogroup tarassovi. N. Z. Vet. J..

[16] Vera E., Taddei S., Cavirani S., Schiavi J., Angelone M., Cabassi C.S., Schiano E., Quintavalla F. (2020). *Leptospira* Seroprevalence in Bardigiano Horses in Northern Italy. Animals.

[17] Bertelloni F., Cilia G., Turchi B., Pinzauti P., Cerri D., Fratini F. (2019). Epidemiology of leptospirosis in North-Central Italy: Fifteen years of serological data (2002–2016). Comp. Immunol. Microbiol. Infect Dis..

[18] Lindtner-Knific R., Vergles-Rataj A., Vlahovic K., Zrimsek P., Dovic A. (2013). Prevalence of antibodies against *Leptospira* sp. in snakes, lizards and turtles in Slovenia. Acta Vet. Scand..

[19] Pleško I., Janovicova E., Lac J. (1964). Contribution to the importance of cold-blooded animals for the circulation of leptospira in nature [in German]. Zentralbl. Bakteriol..

[20] Bharti A.R., Nally J.E., Ricaldi J.N., Matthias M.A., Diaz M.M., Lovett M.A., Levett P.N., Gilman R.H., Willig M.R., Gotuzzo E. (2003). Peru-United States Leptospirosis Consortium. Leptospirosis: A zoonotic disease of global importance. Lancet Infect Dis..

[21] Cosson J.F., Picardeau M., Mielcarek M., Tatard C., Chaval Y., Suputtamongkol Y., Buchy P., Jittapalapong S., Herbreteau V., Morand S. (2014). Epidemiology of Leptospira transmitted by rodents in Southeast Asia. PLoS Negl. Trop. Dis..

[22] Pérez-Flores J., López-Fernáadez O., Atilano D., Garcia-Besné G., Charruau P. (2021). Serum antileptospiral agglutinins in sea turtles (*Eretmochelys imbricata* and *Chelonia mydas*) from the gulf of Mexico. J. Herpetol. Med. Surg..

[23] Abdulla P.K., Karstad L. (1962). Experimental infections with *Leptospira pomona* in snakes and turtles. Zoonoses Res..

[24] Zimmerman L.M., Vogel L.A., Bowden R.M. (2010). Understanding the vertebrate immune system: Insights from the reptilian perspective. J. Exp. Biol..

[25] Zimmerman L.M., Bowden R.M., Vogel L.A. (2013). Red-eared slider turtles lack response to immunization with keyhole limpet hemocyanin but have high levels of natural antibodies. ISRN Zool..

